# Corneal densitometry in high myopia

**DOI:** 10.1186/s12886-018-0851-x

**Published:** 2018-07-25

**Authors:** Jing Dong, Yaqin Zhang, Haining Zhang, Zhijie Jia, Suhua Zhang, Bin Sun, Yongqing Han, Xiaogang Wang

**Affiliations:** 1 Department of Ophthalmology, The First Hospital of Shanxi Medical University, Taiyuan, 030001 Shanxi People’s Republic of China; 2 Department of Ophthalmology, Shanxi Eye Hospital, No. 100 Fudong Street, Taiyuan, 030002 Shanxi People’s Republic of China; 3 Department of Ophthalmology, Affiliated Hospital of Inner Mongolia University for the Nationalities, Tongliao, 028007 Inner Mongolia People’s Republic of China

**Keywords:** Corneal densitometry, High myopia, Scheimpflug, Normal

## Abstract

**Background:**

To investigate corneal densitometry values obtained using Scheimpflug tomography in normal and highly myopic (HM) eyes and to assess the differences in densitometry values between them.

**Methods:**

Highly myopic and normal corneas were examined using the Pentacam Scheimpflug imaging system. Corneal densitometry was automatically performed over a 12-mm diameter area, which was divided on the basis of annular concentric zones (0–2 mm, 2–6 mm, 6–10 mm, 10–12 mm, total diameter) and depth (anterior layer: inner 120 μm; center layer: from 120 μm to the last 60 μm; posterior layer: last 60 μm; total corneal thickness).

**Results:**

A total of 100 normal and 100 HM eyes were enrolled in this study. Upon total corneal thickness densitometry, the HM group was found to have significantly lower values compared with the normal group in 4 annuli, including the 2 mm central zone, 2-6 mm zone, 6–10 mm zone, and 0–12 mm total diameter. Upon anterior layer densitometry, the HM group demonstrated statistically lower values in the 2-6 mm and 6–10 mm zones. Upon densitometry of the central and posterior layers, the HM group was found to have lower values in all annuli.

**Conclusions:**

The densitometry map reveals that light backscatter was lower in most portions of the HM cornea than in the normal cornea.

## Background

High myopia (HM) is characterized by a refractive error greater than − 6.0 diopters. The prevalence of HM is approximately 2.7% worldwide, whereas the prevalence in young Chinese individuals is about 20% [1, 2]. With HM being the fourth leading cause of blindness, 70% of HM eyes have the chance to progress to sight-threatening pathologic retinal impairments, including retinal detachment, retinal degeneration, choroidal neovascularization, and choroidal degeneration [3]. Changes in corneal-related parameters in HM eyes, such as corneal curvature, corneal thickness, and endothelial density, are still under debate [4–7].Previous study of biomechanical properties of cornea demonstrated that corneal hysteresis was significantly lower in HM, which may indicate that some aspects of corneal biomechanical properties such as elasticity, viscosity, hydration, stiffness may be compromised in HM eyes [8].

As a relatively new imaging method, Scheimpflug photography can provide a quantification assessment of light scattering and help assess corneal infiltrates [9]. PentacamHR, a noninvasive, rapid, and reproducible optical system (Oculus GMbH, Wetzlar, Germany), can be used to assess the ocular anterior segment from the anterior corneal surface to the posterior lens surface for corneal topography, corneal pachymetry, anterior chamber depth analysis, and lens clarity analysis [10]. The ability of Pentacam to measure corneal transparency changes objectively and noninvasively may help monitor corneal disease progression and even improve corresponding management.

Corneal densitometry, as an indicator for corneal health, has many applications in corneal diagnosis, such as the diagnosis of bacterial keratitis, keratoconus, pseudoexfoliation syndrome, Fuchs endothelial dystrophy, and rheumatoid arthritis, etc. [11–15] The use of this technique to assess corneal transparency in HM patients has not been reported before, and this technique may provide some useful information when assessing the corneal clarity of HM eyes. The aim of this study was to perform corneal densitometry of normal and HM corneas, to investigate the imaging capabilities of Pentacam in patients with HM and the potential differences in corneal densitometry values between normal and HM eyes.

## Methods

This study was performed at the Shanxi Eye Hospital (Taiyuan, Shanxi, China). The research protocols were approved by the institutional review boards of Shanxi Eye Hospital and were carried out in accordance with the tenets of the Declaration of Helsinki. Written informed consent was obtained from each patient.

### Subjects

We chose Han Chinese subjects to eliminate the possible influences of differences in ethnic groups. The participants with normal and HM eyes were chosen from the Ophthalmic Clinic Center at the Shanxi Eye Hospital. The inclusion criteria for the normal participants were as follows: best-corrected visual acuity (BCVA) of ≥16/20, a refractive error < 5 diopter (D) spheres and IOLMaster axial length (AL) less than 25 mm, normal slit-lamp and fundoscopy results, intraocular pressure (IOP) < 21 mmHg, and no history of ocular or systemic corticosteroid use. The inclusion criteria for the participants with HM were as follows: BCVA ≥20/40, spherical refractive error more negative than − 6 diopters, IOLMaster axial length longer than 25 mm, and central fixation sufficiently stable for image capture. Participants with keratoconus; previous corneal lesions; prior surgery of the cornea; severe cataracts; glaucoma; posterior abnormalities, such as choroidal neovascularization, retinoschisis, retinal detachment, or macular holes; and missing data were excluded.

### Data acquisition

Corneal densitometry was performed using the rotating Scheimpflug anterior segment analyzer (Pentacam HR, Oculus GMbH, Wetzlar, Germany). The participant was asked to place his/her chin on the chin rest and press his/her forehead against the forehead strap. The participant’s eye was aligned to the visual axis with a central fixation target. After proper alignment and blinking a few times, the automatic release mode, with 25 single Scheimpflug images, was started for each eye. Only cases with acceptable image quality were included in the final analysis. A single trained operator performed all examinations. All parameters were automatically calculated by the Pentacam software (Version 1.20r36).

**Fig. 1:**
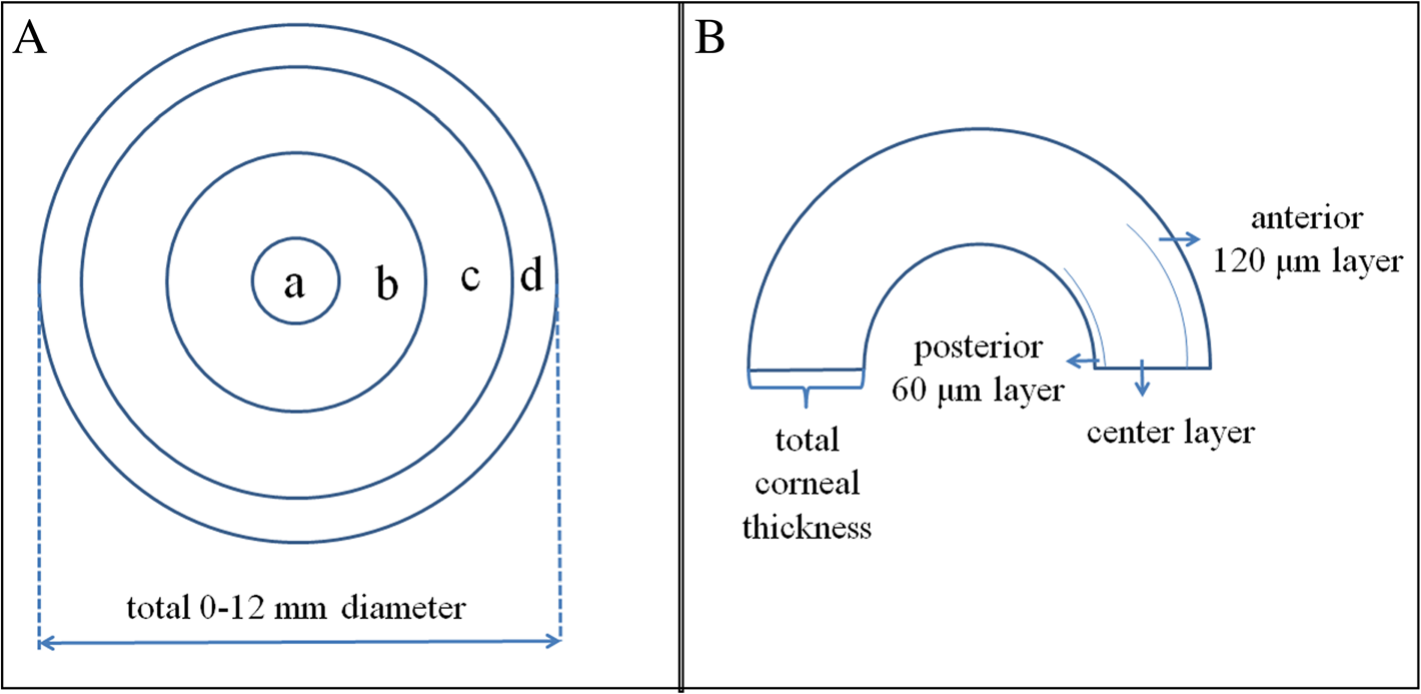
Schematic diagram of standard corneal densitometry analysis using Pentacam. Panel A: densitometry analysis of concentric annular zones centered at the corneal apex (a: 0–2 mm, b: 2–6 mm, c: 6–10 mm, d: 10–12 mm); Panel B corneal densitometry analysis based on different depths (anterior layer: inner 120 μm; center layer: from 120 μm to the last 60 μm; posterior layer: last 60 μm; total corneal thickness)

**Fig. 2:**
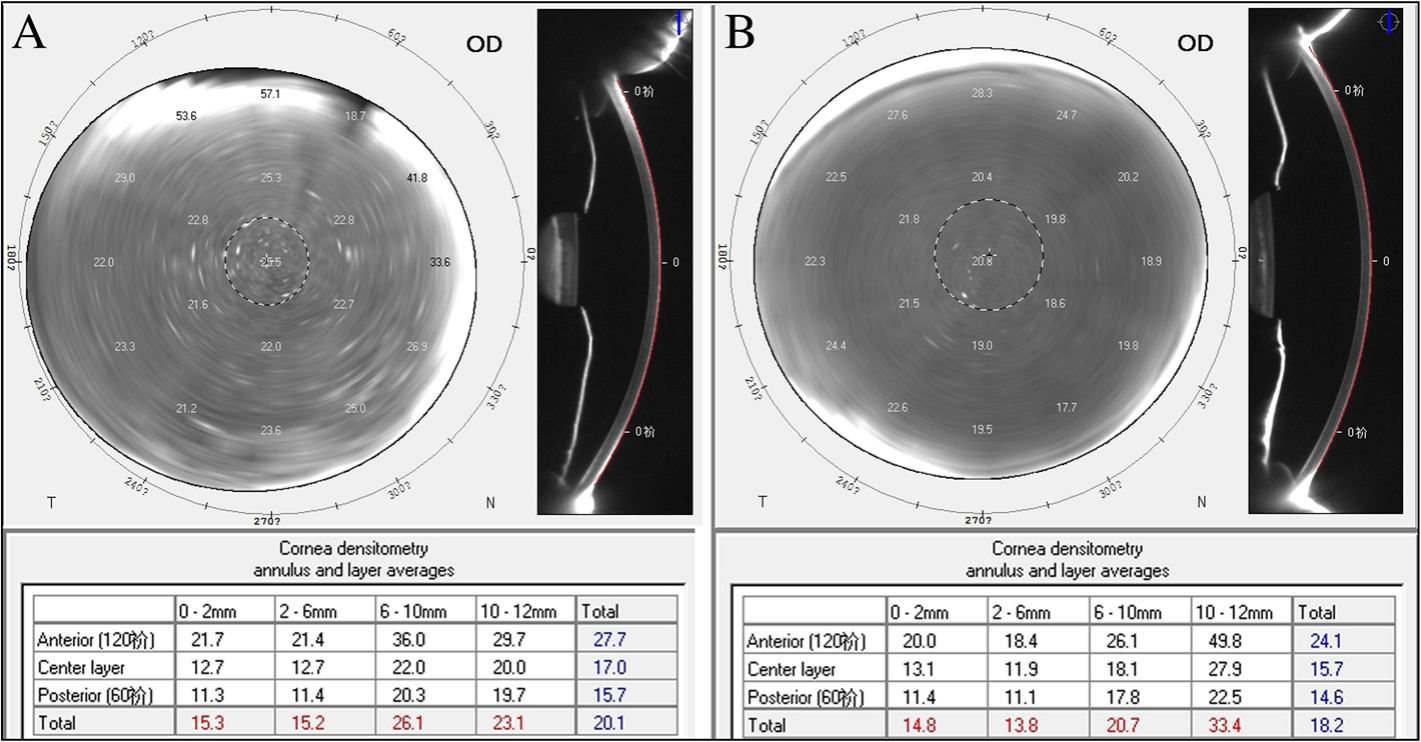
The Scheimpflug tomography images and corresponding corneal densitometry values of a normal eye (Panel **a**) and a highly myopic eye (Panel **b**)

An internal standard corneal densitometry analysis software measures the backscattered light over a 12-mm diameter corneal area (Fig. 1). Zonal corneal densitometry values were automatically measured in 4 concentric annular zones centered at the apex of the cornea (0–2 mm, 2–6 mm, 6–10 mm, 10–12 mm, total diameter) and by depth (anterior layer: inner 120 μm; center layer: from 120 μm to the last 60 μm; posterior layer: last 60 μm; total corneal thickness; Fig. 2).

### Statistical analysis

Statistical analysis was performed using SPSS ver. 13.0. The statistical significance of the intergroup differences in age and axial length was evaluated using the independent sample t-test. The Mann-Whitney U test was used to assess the differences in corneal densitometry values between the two groups. Corneal densitometry values and age were correlated using Pearson’s bivariate regression. The significance level for all of the tests was set at 5%.

## Results

A total of 200 participants were included in the study: 100 participants with normal corneas and 100 participants with HM corneas. There were 44 female and 56 male participants in the normal group and 42 female and 58 male participants in the HM group. No significant sex differences were found between the two groups (*P* = 0.435). The mean age in the normal group was 65 ± 10 years (range, 20–87 years), which was not significantly higher than that in the HM group 62 ± 13 years (range, 18–92 years; *P* = 0.086). The AL in the normal group was 23.08 ± 0.79 mm (range, 21.76–24.83 mm), which was statistically shorter than that in the HM group, that is, 28.79 ± 2.52 mm (range, 25.03–35.12 mm; *P* < 0.001).

Total corneal light backscatter was significantly lower in the HM group (HM = 18.9 ± 4.4, normal = 20.3 ± 4.3, *P* = 0.008) than in the normal group. Considering the densitometry values for the total corneal thickness, which was separated by concentric annular zones around the apex, the corneal light backscatter in the first 3 annuli (center to 2 mm, 2–6 mm diameter, and 6–10 mm diameter) was statistically lower in the HM group than in the normal group (HM = 14.7 ± 1.5, normal = 15.2 ± 1.6, *P* = 0.028; HM = 14.1 ± 2.1, normal = 15.4 ± 3.9, *P* = 0.001; HM = 22.1 ± 8.8, normal = 25.2 ± 8.0, *P* = 0.002, respectively). However, no significant difference was found in the 10–12 mm diameter annular zone between the two groups (*P* = 0.203).

**Fig. 3:**
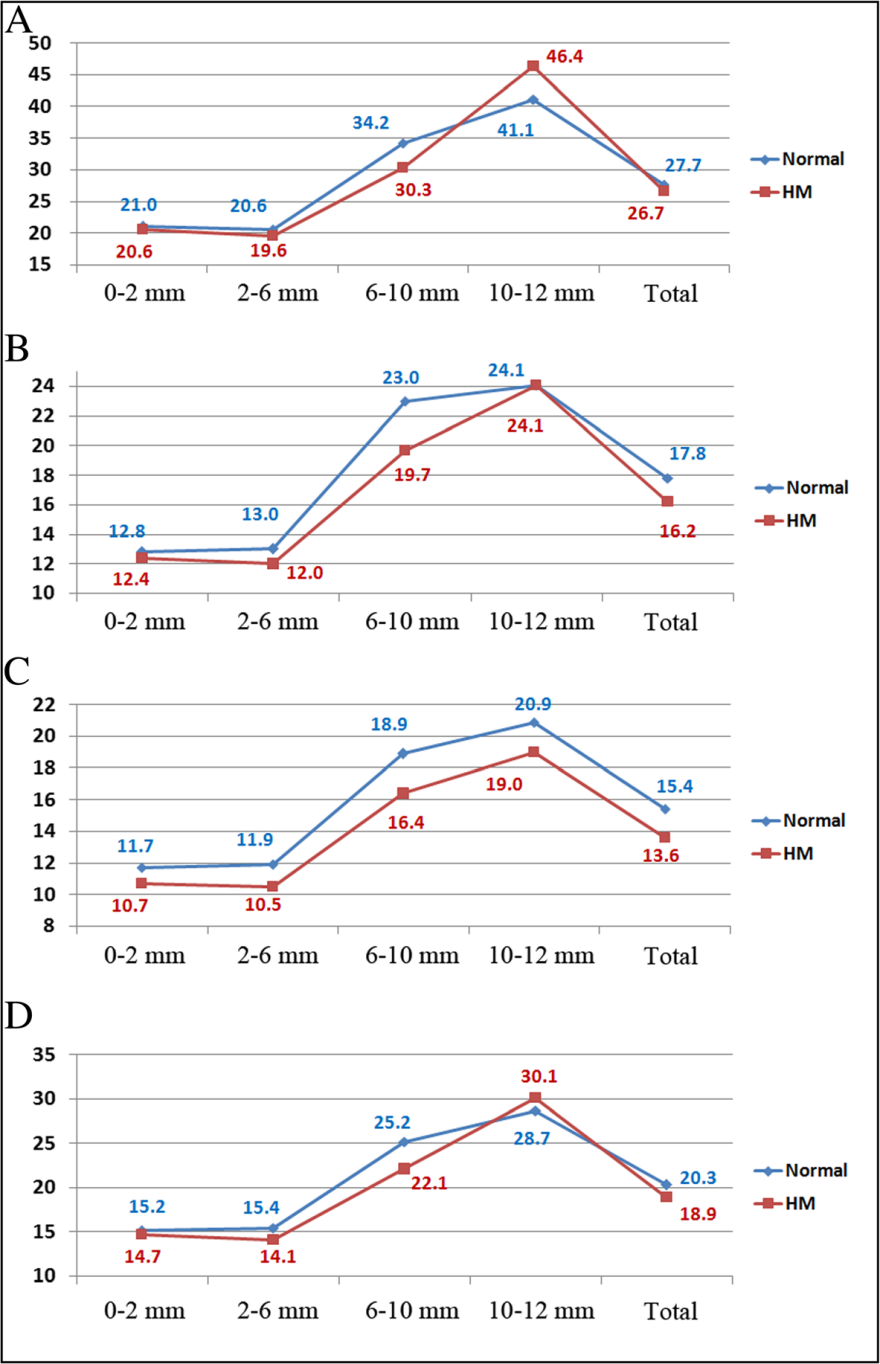
Mean densitometry values of the anterior layer (panel **a**), center layer (panel **b**), posterior layer (panel **c**), and total corneal thickness (panel **d**) in the normal and HM groups. HM = high myopia; Total = total 0–12 mm diameter

When the cornea was divided into 3 layers, densitometry of the anterior layer of the concentric annular zones in the first 3 annuli and the total 0–12 mm diameter revealed the same tendency of a lower backscatter in the central corneal in the HM group. However, the anterior layer densitometry values of the 10–12 mm annular zone were higher in the HM group than in the normal group (HM = 46.4 ± 14.2, normal = 41.1 ± 13.5, *P* = 0.004). Upon densitometry of the central and posterior layers, all concentric annular zones were found to have a lower light backscatter in the HM group than in the normal group. These results are summarized in Fig. 3 and Table 1.

**Table 1:** Corneal densitometry values of the normal and highly myopic groups

	Normal Group (*n* = 100)	HM Group (*n* = 100)	Difference	P*
Anterior 120 μm				
0–2 mm	21.0 ± 2.7	20.6 ± 2.2	0.4 ± 0.3	0.474
2–6 mm	20.6 ± 3.3	19.6 ± 2.9	1.1 ± 0.4	**0.017**
6–10 mm	34.2 ± 11.6	30.3 ± 13.4	3.9 ± 1.8	**0.004**
10–12 mm	41.1 ± 13.5	46.4 ± 14.2	− 5.3 ± 2.0	**0.004**
Total 0–12 mm	27.7 ± 6.1	26.7 ± 6.3	1.0 ± 0.9	0.143
Center layer				
0–2 mm	12.8 ± 1.2	12.4 ± 0.9	0.4 ± 0.1	**0.030**
2–6 mm	13.0 ± 2.2	12.0 ± 1.7	1.0 ± 0.3	**< 0.001**
6–10 mm	23.0 ± 8.0	19.7 ± 8.3	3.3 ± 1.2	**0.001**
10–12 mm	24.1 ± 7.1	24.1 ± 6.6	0 ± 1.0	0.823
Total 0–12 mm	17.8 ± 4.2	16.2 ± 3.9	1.6 ± 0.6	**0.002**
Posterior 60 μm				
0–2 mm	11.7 ± 1.9	10.7 ± 1.4	1.1 ± 0.2	**< 0.001**
2–6 mm	11.9 ± 2.3	10.5 ± 1.5	1.4 ± 0.3	**< 0.001**
6–10 mm	18.9 ± 4.9	16.4 ± 5.3	2.5 ± 0.7	**< 0.001**
10–12 mm	20.9 ± 5.5	19.0 ± 5.2	1.9 ± 0.8	**0.009**
Total 0–12 mm	15.4 ± 3.0	13.6 ± 2.8	1.7 ± 0.4	**< 0.001**
Total corneal thickness				
0–2 mm	15.2 ± 1.6	14.7 ± 1.5	0.5 ± 0.2	**0.028**
2–6 mm	15.4 ± 3.9	14.1 ± 2.1	1.3 ± 0.4	**0.001**
6–10 mm	25.2 ± 8.0	22.1 ± 8.8	3.1 ± 1.2	**0.002**
10–12 mm	28.7 ± 7.9	30.1 ± 8.0	−1.4 ± 1.1	0.203
Total 0–12 mm	20.3 ± 4.3	18.9 ± 4.4	1.4 ± 0.6	**0.008**

*HM* high myopia; * Mann-Whitney u test for statistical analysis; numbers in bold are statistically significant results

The densitometry values of the total corneal thickness were significantly correlated with age in both the normal and HM group (*r* = 0.540, *P* < 0.001; *r* = 0.711, P < 0.001). Moreover, the densitometry values of the anterior layer, center layer, and posterior layer were all correlated well with age in both groups (normal: *r* = 0.504, *r* = 0.520, *r* = 0.570; HM: *r* = 0.678, *r* = 0.721, *r* = 0.747; all P < 0.001). Central corneal thickness was positively correlated with total corneal densitometry values in HM group (*r* = 0.198,*P* = 0.048) but not in the normal group (*r* = 0.135, *P* = 0.181). No significant correlation was found between AL, mean keratometry and total corneal densitometry values in both groups (all *P* > 0.05).

## Discussion

In previous studies, HM eyes were reported to have different cornea-related parameters, such as corneal curvature, corneal thickness, corneal endothelial density, corneal hysteresis, and corneal resistance factor, compared with normal eyes [4, 5, 8, 16–19]. Clinical analysis of corneal light backscatter played an important role in the evaluation of the corneal status and in monitoring the progression of some corneal diseases [11–15]. In our study, we found a decrease in light backscatter in the central cornea (10 mm in diameter) and total diameter in the HM group. A lower densitometry in the central cornea was also seen in the anterior, center, and posterior layers.

As demonstrated in a previous study, corneal densitometry provides useful information about corneal clarity [14]. In a normal cornea, the anterior superficial epithelium, stromal, and the posterior corneal endothelium contribute to the total corneal densitometry value. In the HM group, decreased corneal densitometry values were found for both the total corneal thickness and the 3 seperated layers. This changing tendency may be attributed to several factors: 1) Corneal biomechanical property changes in HM. The study by Shen et al. showed that corneal hysteresis (CH) was significantly lower in HM corneas [8]. CH is a parameter related to momentary deformation of the cornea. In the current study, participants with HM corneas had lower corneal densitometry values. However, corneal damage could not be proven. We hypothesize that lower densitometry values, as well as lower CH, indicate that some biomechanical properties of the cornea have been altered. 2) Corneal endothelial cell density changes in low and moderate myopic eyes. Delshad et al. demonstrated that the more myopic eyes tend to have a lower endothelial cell density and cell hexagonality [16]. As endothelial cells are a part of the light scattering corneal tissue, the lower endothelial cell density may result in lower corneal densitometry values. Our present study showed significantly lower corneal densitometry values for the posterior layer in HM eyes, which confirms the corneal endothelial cell density changes. 3) Potential changes in the structures inside the cornea, such as nerves, cell nuclei, the spacing of the corneal fibrils, size of the collagen fibrils, the extracellular matrix surrounding the collagen fibrils, and corneal hydration. Previous research has demonstrated that changes in the above-mentioned corneal structures play an important role in corneal transparency [20–22]. Therefore, our findings on the lower corneal densitometry values in HM eyes may be correlated with these structural changes. However, more cellular and histological studies are needed to prove these potential changes in HM corneas.

In the periphery (10–12 mm diameter), either no significant difference was found between the two groups or higher corneal densitometry values were found in the HM group than in the control group. Because of the low repeatability and reproducibility of peripheral densitometry measurements reported a previous normative study, this result has to be interpreted with caution in clinical practice [23]. Moreover, the white-to-white distance variation, which may lead to the inclusion of some proportion of the limbus and sclera in automatic corneal densitometry analysis, could mistakenly cause higher corneal densitometry values [13]. A recent study demonstrated that corneal densitometry values increased with age in Spanish patients [24]. Our study confirmed this correlation.

Similar to a previous study, neither mean keratometry readings nor central corneal thickness was correlated with total corneal densitometry values in the normal group in this study [24]. However, the positive correlation between central corneal thickness and total corneal densitometry values was found in the HM group. This may be attributed to the relatively less endothelial cell density with myopic increasing, which has been demonstrated in a previous study [16].

A previous study has shown that participants of different races, even different subpopulations in the same race, may have different cornea-related parameters [25]. The current study involved a limited number of eyes and only included Chinese participants; therefore the results cannot be directly generalized to different ethnic backgrounds. Despite the above-mentioned limitation, this prospective study to investigate the corneal densitometry values in HM eyes provides useful information for clinical practice.

## Conclusions

In conclusion, the Scheimpflug densitometry map revealed that light backscatter was lower in most portions of a HM cornea than in the normal cornea. Moreover, age was positively correlated with the total corneal densitometry values in both groups. Therefore, corneal densitometry may play a valuable role in characterizing HM corneas.

## Abbreviations

AL: Axial length
BCVA: Best-corrected visual acuity
CH: Corneal hysteresis
D: Diopter
HM: High myopia
IOP: Intraocular pressure
